# Estrogen inhibits endoplasmic reticulum stress and ameliorates myocardial ischemia/reperfusion injury in rats by upregulating SERCA2a

**DOI:** 10.1186/s12964-022-00842-2

**Published:** 2022-03-24

**Authors:** Jingwen Chen, Yang Liu, Defeng Pan, Tongda Xu, Yuanyuan Luo, Wanling Wu, Pei Wu, Hong Zhu, Dongye Li

**Affiliations:** 1 Institute of Cardiovascular Disease Research, Xuzhou Medical University, 84 West Huaihai Road, Xuzhou, 221002 Jiangsu People’s Republic of China; 2 Department of Cardiology, The Affiliated Hospital of Xuzhou Medical University, 99 West Huaihai Road, Xuzhou, 221002 Jiangsu People’s Republic of China

**Keywords:** Myocardial ischemia/reperfusion injury, Estrogen, Endoplasmic reticulum stress, SERCA2a

## Abstract

**Background:**

The incidence of coronary heart disease (CHD) in premenopausal women is significantly lower than that of men of the same age, suggesting protective roles of estrogen for the cardiovascular system against CHD. This study aimed to confirm the protective effect of estrogen on myocardium during myocardial ischemia/reperfusion (MI/R) injury and explore the underlying mechanisms.

**Methods:**

Neonatal rat cardiomyocytes and Sprague–Dawley rats were used in this study. Different groups were treated by bilateral ovariectomy, 17β-estradiol (E2), adenoviral infection, or siRNA transfection. The expression of sarcoplasmic reticulum Ca^2+^ ATPase pump (SERCA2a) and endoplasmic reticulum (ER) stress-related proteins were measured in each group to examine the effect of different E2 levels and determine the relationship between SERCA2a and ER stress. The cell apoptosis, myocardial infarction size, levels of apoptosis and serum cardiac troponin I, ejection fraction, calcium transient, and morphology changes of the myocardium and ER were examined to verify the effects of E2 on the myocardium.

**Results:**

Bilateral ovariectomy resulted in reduced SERCA2a levels and more severe MI/R injury. E2 treatment increased SERCA2a expression. Both E2 treatment and exogenous SERCA2a overexpression decreased levels of ER stress-related proteins and alleviated myocardial damage. In contrast, *SERCA2a* knockdown exacerbated ER stress and myocardial damage. Addition of E2 after *SERCA2a* knockdown did not effectively inhibit ER stress or reduce myocardial injury.

**Conclusions:**

Our data demonstrate that estrogen inhibits ER stress and attenuates MI/R injury by upregulating SERCA2a. These results provide a new potential target for therapeutic intervention and drug discovery in CHD.

**Video Abstract**

**Supplementary Information:**

The online version contains supplementary material available at 10.1186/s12964-022-00842-2.

## Background

Myocardial ischemia/reperfusion (MI/R) injury refers to the increased damage to cardiomyocytes and their resultant functional decrease after the recovery of blood supply, which caused no-reflow, severe arrhythmia disorders, and other myocardial defects [1]. Thus, coronary revascularization induces MI/R injury, which significantly influences the prognosis of coronary heart disease (CHD) patients. Therefore, we need to fully understand the mechanisms of MI/R injury to develop preventative measures.

There are dramatic gender differences in the incidence of CHD [2]; the prevalence of CHD in premenopausal women is low and symptoms usually occur a full decade later than in men [3], suggesting that female hormones, especially estrogen, may play an essential role in prevention of CHD. In the past 20 years, research into cardiovascular disease in women has increased our understanding of the factors that drive female CHD. Estrogen has several effects on cardiovascular function and disease: it modulates vascular function, the inflammatory response, metabolism, insulin sensitivity, cardiac myocytes, stem cell survival, and the development of hypertrophy [4]. It was observed that the female myocardium has stronger resistance to I/R injury in mice, rats, rabbits and, dogs [5, 6]. Young adult female rats exhibit better recovery of contractile function and fewer arrhythmias during reperfusion than age-matched males [7, 8]. Estrogen pretreatment was indicated to exert protective effects against I/R injury on the myocardium [9–12]. In addition, estrogen derivatives 7 has a cardioprotective effect on calcium channels activation and decreasing myocardial necrosis after I/R [13]. Besides, estrogen was also demonstrated to protect against I/R injury of the brain [14], kidney [15], intestine [16], and liver [17].

The mechanisms of MI/R injury primarily include endoplasmic reticulum (ER) stress, oxidative stress, calcium overload, mitochondrial dysfunction, apoptosis, autophagy activation, and epigenetic changes [18]. Among them, ER stress plays an important role. ER is where many proteins are translated and folded. Approximately 33% of transcripts are synthesized in ER, where they are folded and assembled with the assistance of molecular chaperones and oxidoreductases [19]. Various stimuli, such as oxidative stress, ischemia, and Ca^2+^ disorders, can cause unfolded or misfolded proteins to accumulate in the ER. This is termed ER stress, and ER stress signaling triggers the unfolded protein response (UPR) to maintain ER stability. The main pathways of the UPR are activated when GRP78 dissociates from the ER transmembrane sensors proteins. These sensors are inositol-requiring enzyme 1α (IRE1α), protein kinase RNA-like ER kinase (PERK), and activating transcription factor (ATF) 6.

Nevertheless, under persistent or severe ER stress, the UPR will induce apoptosis [20]. The UPR pathway mediated by ATF6 and PERK, which increase expression of the transcription factor CCAAT/enhancer-binding protein homologous protein (CHOP). CHOP inhibits expression of the anti-apoptotic proteins Bcl-2 and Bnip3 in cardiomyocytes. ER stress can also activate caspase 12, which activates caspase 9/3 to mediate non-mitochondrial apoptosis [21].

Estrogen can inhibit ER stress-induced apoptosis in human endometrial cells, pancreatic β cells, and osteoblasts [22–24]. Many studies have investigated the effects of estrogen on ER stress in tumors and neurological diseases [25–27], but few have studied its impacts on cardiovascular diseases.

Sarcoplasmic reticulum Ca^2+^ ATPase pump (SERCA2a) is the primary protein involved in the excitation–contraction coupling of cardiomyocytes. SERCA2a pumps Ca^2+^ from cytoplasm into sarcoplasmic reticulum (SR) in an ATP-dependent manner against the concentration gradient, promoting myocardial relaxation and ensuring enough Ca^2+^ is present in the SR for release. This allows the myocardium to contract normally. Decreased expression or dysfunction of SERCA2a weakens the ability of the SR to uptake Ca^2+^, altering intracellular Ca^2+^ homeostasis [28], which is one of the leading causes of ER stress. Knocking out *SERCA2a* from mouse cardiomyocytes leads to ER stress and apoptosis [29]; conversely expressing exogenous *SERCA2a* in porcine myocardial ischemia reduces ER stress [30]. Our group has previously published that SERCA2a expression is significantly reduced in MI/R injury and the increase of SERCA2a could effectively relieve the myocardium damage [31].

Estrogen acts on a variety of tissues and cell, and also has a significant impact on SERCA2a expression in cardiomyocytes. A previous study showed that SERCA2a expression was significantly reduced in cardiomyocytes after rats underwent bilateral ovariectomy (OVX) [32] but increased in H9c2 cells cultured with 17β-estradiol (E2) [33].

Based on these findings, we propose the following hypothesis: (1) estrogen can inhibit ER stress during MI/R injury and reduce myocardial damage; (2) part of this effect of estrogen on ER stress in cardiomyocytes is achieved by increasing SERCA2a levels to maintain intracellular Ca^2+^ homeostasis. In this study, we will test this hypothesis by detecting SERCA2a expression, ER stress levels, and the degree of MI/R injury.

## Methods

### Cells and culture conditions

Neonatal rat cardiomyocytes (NRCMs) were isolated from the ventricular myocardium of 1-to-3-day-old Sprague–Dawley (SD) rats by enzymatic dissociation. The Animal Center of Xuzhou Medical University (Xuzhou, China) provided neonatal SD rats. The experimental procedures were approved by the Xuzhou Medical University Animal Ethics Committee (permit number: CMCACUC 2018-04-161).

### Animals

Female SD rats (8–10 weeks old) weighing 200–220 g were purchased from the Animal Center of Xuzhou Medical University. The Animal Ethics Committee of Xuzhou Medical University approved all experimental protocols. Rats were housed four per cage under controlled temperature (24 ± 1 °C) and humidity (55% ± 5%) conditions, with luminosity cycles of 12-h light/12-h dark and free access to food and water.

### In vitro assays

#### Cell culture, E2 administration, adenoviral infection, and siRNA transfection

The ventricular myocardium of 1-to-3-day-old SD rats was cut into pieces on ice. The myocardium tissue was then shredded and incubated in 0.08% type I collagenase and 0.08% trypsin for digestion. The digestion product was filtered and collected after 5–6 repetitions. Suspension cells were then gathered and centrifuged at 1000×*g* for 5 min. The cells were then resuspended in DMEM (Dulbecco's modified eagle medium) containing 20% charcoal-stripped FBS (fetal bovine serum) and 100 μg/mL penicillin/streptomycin and cultured in 5% CO_2_ for 90 min at 37 °C. Unwalled myocardial cell suspension were then transferred to 3-cm cell dishes at a density of 5 × 10^5^ cells/mL and cultured in 5% CO_2_ at 37 °C.

After 48 h, Adenovirus-mediated gene transfer was implemented as previously described [34]. Briefly, NRCMs were infected with adenoviral vectors containing the *Atp2a2* gene or *EGFP* (Ad-SERCA2a and Ad-EGFP, respectively, Hanbio Biotechnology Co., Ltd, Shanghai, China). Ad-EGFP was used as a control, and both constructs were used at a multiplicity of infection (MOI) of 200 pfu. Then replace the culture medium 6 h later.

The siRNA transfection and adenoviral infection were performed simultaneously. *SERCA2* siRNA and scramble RNA were purchased from GenePharma (Shanghai, China) and transfected into NRCMs using X-tremeGENE™ siRNA Transfection Reagent (Sigma-Aldrich) following the manufacturer's protocol. The *SERCA2* siRNA sequence is as follows: sense, 5′-GACUUACUAGUUAGAAUUUTT-3′, antisense, 5′-AAAUUCUAACUAGUAAGUCUU-3′. Transfected cells were cultured for 48 h before performing in vitro hypoxia/reoxygenation (H/R) assays.

After 12 h of siRNA transfection and adenoviral infection, E2 (Sigma-Aldrich, St. Louis, MO, USA) dissolved in DMSO (dimethyl sulfoxide) was added to the culture medium of the E2 intervention groups at a final concentration of 10 nM. The total acting time of E2 was approximately 36 h.

#### The H/R protocol

Hypoxia conditions were produced by culturing cells with D-Hanks solution (5.37 mM KCl, 136.89 mM NaCl, 0.44 mM KH_2_PO_4_, 0.338 mM Na_2_HPO_4_, 4.166 mM NaHCO_3_, and 5 mM D-glucose, pH 7.3–7.4) at 37 °C, saturated with 95% N_2_ and 5% CO_2_. The pH was maintained at 6.8 with lactate to mimic an ischemic solution. The cells were cultured for 4 h in a hypoxic incubator equilibrated with 1% O_2_, 5% CO_2_, and 94% N_2_. After hypoxia treatment, the culture medium was replaced with fresh DMEM containing 10% FBS for another 4 h to initiate reoxygenation.

#### Flow cytometry

The Annexin V-APC Apoptosis Detection Kit (KeyGen Biotech, Nanjing, China) was used to detect apoptosis. Briefly, 5 × 10^5^ cells were collected, washed twice with phosphate buffered saline (PBS), resuspended in 500 μL binding buffer and incubated with 5 μL Annexin V-APC and 5 μL propidium iodide (PI) for 10 min before analysis using a FACS Calibur flow cytometer (BD Biosciences, Franklin Lakes, NJ, USA).

#### Measurements and analysis of calcium transient

NRCMs were plated on coverslips in 3-cm dishes for routine cultivation. After carrying out all intervention protocols, NRCMs were eluted with Hanks Balanced Salt Solution (HBSS), and then incubated with 5 μM/mL Fura 2-AM (Dojindo Laboratories, Kumamoto Japan) in DMSO and diluted in HBSS for 30 min at 37 °C followed by another HBSS elution. NRCMs on coverslips were then sent for calcium transient recording using a dual-excitation fluorescence photomultiplier system (IonOptix, Milton, MA, USA). Briefly, the NRCMs were continuously covered with warm oxygenated Tyrode's solution (140 mM NaCl, 1 mM MgCl_2_, 6 mM KCl, 10 mM glucose, 2 mM CaCl_2_, and 5 mM HEPES, pH 7.4) [35]. Fluorescence intensity at 510 nm was detected with alternate scanning by fluorescence emissions at 340 nm and 380 nm. Intracellular Ca^2+^ levels and the intracellular Ca^2+^ transient decay time constant were recorded and analyzed by the SoftEdge MyoCam® system (IonOptix).

### In vivo assays

#### OVX protocol and E2 administration

First, 8–10-week-old rats underwent OVX as previously described [36]. Briefly, rats were anesthetized with isoflurane and fixed in the prone position. The hair between the ribs and pelvis at the bottom of the back was shaved and the area disinfected. An approximately 1 cm incision was made parallel to the spine on the right side of the disinfected area. Then the muscle was separated, and small pink spherical ovaries appeared behind the kidney. The ovarian artery and fallopian tube under the ovary were then ligated, the ovaries were removed and the muscle and skin was sutured after resetting the abdominal organs. These steps were then repeated to remove the left ovary. In the sham operation group, only a small piece of adipose tissue from both sides of the incision was removed. Rats were given E2 (dissolved in olive oil) by subcutaneous injection (40 μg/kg/day) for 31 consecutive days starting 2 weeks after the sham operation or bilateral OVX.

#### Adenoviral infection and MI/R protocol

The *Atp2a2* gene or *EGFP* (Hanbio Biotechnology Co., Ltd) were transferred into the heart by intramuscular injections. First, the left thorax was opened to expose the heart. There were three injection sites in the left ventricle free wall with a total volume of 15 uL of Ad-SERCA2a or Ad-EGFP (1 × 10^10^ pfu). Three days after transfection, the hearts underwent MI/R, which was induced by left anterior descending artery (LAD) ligation for 30 min followed by 24 h reperfusion.

#### Terminal deoxynucleotidyl transferase dUTP-nick end labeling (TUNEL)

TUNEL staining was performed according to the manufacturer's protocol (Beyotime Institute of Biotechnology, Shanghai, China). Briefly, after fixation in a 4% paraformaldehyde solution for 15 min, tissue slides were washed twice with PBS, covered with 20 µg/mL proteinase K without DNase for 25 min, and then with the TUNEL reaction mixture containing terminal deoxynucleotidyl transferase and fluorescein isothiocyanate-dUTP. The samples were analyzed in the range of 520 ± 20 nm (green) and 460 nm (blue) under a fluorescent and UV light microscope (Tokyo, Japan). For each group, five random fields were examined.

#### Evans blue and 2,3,5-triphenyltetrazolium chloride (TTC) staining

Evans blue and TTC (Sigma-Aldrich) staining were used to show the myocardial infarct size. After 24 h of reperfusion, the LAD was ligated again in the position of the previous ligation. Then the aorta was clamped, and 1.5 mL of 2% Evans blue was injected into the left atrium to stain the left ventricle myocardium. After 30 s, 10% KCl was injected to cause cardiac arrest. The heart was quickly removed and frozen for 10 min at − 80 °C, and then cut into five 2-mm thick slices. The myocardial slices were immersed in 1% TTC solution (preheated to 37 °C) for 30 min. The color of the non-ischemic area was blue, the white area was the infarct area, and the white/red area was the area at risk (AAR).

#### Enzyme-linked immunosorbent assay (ELISA)

To measure cardiac troponin‑I (cTn I) levels in the serum of each group, ELISA kits for cTn I were purchased from KeyGEN Bio tech. A multi-detection microplate reader (Synergy™HT, BioTek, Winooski, VT USA) was used for detection. All protocols were performed following the kit's manufacturing recommendations.

#### Echocardiography

Transthoracic echocardiography was used to measure the ejection fraction (EF%) of the rat heart and to observe the movement of left ventricular wall. The rats were anesthetized after 24 h of reperfusion, and the hair on their left front chest wall was removed. A Vevo 770 (Visual Sonics, Toronto, Canada) with a 13-MHz probe was used to performed this study.

#### Hematoxylin–eosin (HE) staining

HE staining was used to observe pathological changes to myocardial tissue. Paraffin sections of myocardial tissue were deparaffinized and then placed in a hematoxylin solution for staining. The sections were rinsed with running water for 1 h after color separation. Then the sections that turned blue after dehydration were stained in an eosin staining solution for 5 min. The samples were dehydrated again and soaked in xylene. The sections were then covered with cover glass and observed under an optical microscope.

#### Masson staining

Masson staining was used to identify fibrosis within the myocardial tissue. After dewaxing, paraffin sections of myocardial tissue were stained with Weigert's hematoxylin solution for 5–10 min, and then stained with Masson blue and ponceau magenta staining solutions for 5 min. The tissues were then placed into aniline blue for 2 min after washing with a phosphomolybdic acid solution. Finally, the sections were dehydrated, made transparent with xylene, and covered. Nuclei and collagen fibers were blue, while the red area was the cytoplasm, muscles, and erythrocytes.

#### Transmission electron microscopy (TEM)

Left ventricular tissues were fixed in 2.5% glutaraldehyde, and then dehydrated with graded ethanol and acetone solution. The tissues were then embedded and solidified, cut into 50–60 nm slices with an ultramicrotome (Leica EM UC7rt A-1170, Wetzlar, Germany), and then double-stained with 3% uranyl acetate-lead citrate. Finally, the samples were scanned under a TEM (Tecnai™ G2 Spirit TWIN, FEI Co., Hillsboro, Oregon, USA).

### Quantitative real-time PCR (qRT-PCR)

Total RNA was extracted from isolated myocardial tissue using TRIzol reagent (Invitrogen, Carlsbad, CA, USA). Then, 2 μg RNA samples from each group were assayed by qRT-PCR using the SuperReal PreMix Color kit (TIANGEN Biotech Co. Ltd., Beijing, China) and an ABI7500 real-time DNA detection system (Applied Biosystems, Waltham, MA, USA). Raw data were normalized to *GAPDH*, and relative expression was calculated using the 2^−ΔΔCT^. The primers used for qRT-PCR were as follows:

**Table:** 

SERCA2a	Forward: 5′-GTCTGGGTTGTCCTCCTTA-3′
	Reverse: 5′-TTTTCCGTTACCTGGCTAT-3′
GRP78	Forward: 5′-GTGCGGGCGGAGGAGGAG-3′
	Reverse: 5′-ATACGACGGTGTGATGCGGTTG-3′
CHOP	Forward: 5′-GTCACAAGCACCTCCCAAAGCC-3′
	Reverse: 5′-CGCACTGACCACTCTGTTTCCG-3′
Calreticulin (CRT)	Forward : 5′-CGCACTGACCACTCTGTTTCCG-3′
	Reverse: 5′-AATCGGGCATCTTGGCTTGTCTG-3′
GAPDH	Forward: 5′-GAGAGGGAAATCGTGCGT-3′
	Reverse: 5′-GGAGGAAGAGGATGCGG-3′

### Western blotting

Myocardial tissues were lysed to extract protein, which was subjected to SDS–polyacrylamide gel electrophoresis, and then transferred to polyvinylidene fluoride membrane (Merck Millipore, Billerica, MA, USA). Western blotting was performed using primary antibodies against SERCA2a (1:20,000; Abcam, Cambridge, UK), GRP78 (1:10,000), CRT (1:1000), CHOP (1:500), GAPDH (1:10,000; all from Proteintech, Wuhan, China), Bcl-2 (1:1000), Bax (1:1000; both from Cell Signaling Technology, Danvers, MA, USA), and caspase 12 (1:1000; Affinity Biosciences, Inc., Cincinnati, OH, USA). Anti-mouse- and anti-rabbit-horseradish peroxidase-conjugated (HRP) secondary antibodies were purchased from Proteintech. Immunoreactive bands were visualized with ECL chemiluminescent HRP substrate (Bioworld Technology, Inc., Bloomington, MN, USA) using a chemiluminescence detection system (Tanon Imaging System, Tanon, Shanghai, China).

### Statistical analysis

Data are expressed as mean ± SEM. All statistical analyses were conducted with GraphPad Prism version 6.0 (GraphPad Software, Inc., San Diego, CA, USA). For statistical analyses, one-way ANOVA was used for intergroup comparison, followed by Bonferroni's multiple comparisons exact probability test. *P* < 0.05 was considered statistically significant.

## Results

### E2 increased SERCA2a expression

**Fig. 1:**
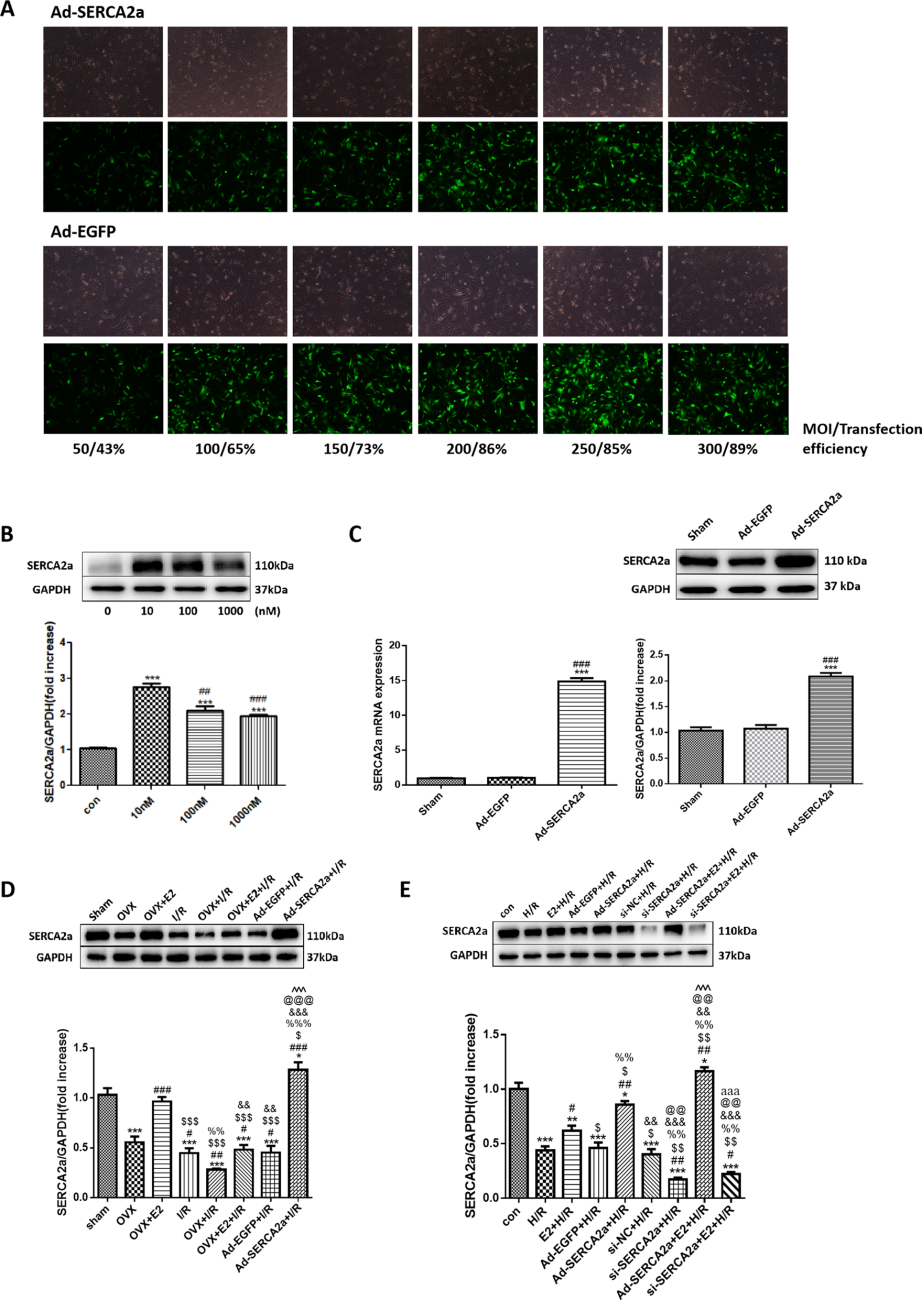
The efficiency of SERCA2a overexpression. E2 increased SERCA2a expression in NRCMs. **a** MOI of Ad-SERCA2a and Ad-EGFP in NRCMs. **b** The effects of different concentrations of E2 solution on SERCA2a expression in vitro (n = 3; ***P < 0.001 vs. con; ^##^*P* < 0.01, ^###^ *P* < 0.001 vs. 10 nM). **c** The mRNA and protein expression of SERCA2a after Ad-SERCA2a or Ad-EGFP infection (n = 5; ***P < 0.001 vs. Sham; ^###^*P* < 0.001 vs. Ad-EGFP). **d** SERCA2a protein expression in vivo (n = 3; *P < 0.05, ***P < 0.001 vs. sham; ^#^*P* < 0.05, ^##^*P* < 0.01, ^###^*P* < 0.001 vs. OVX; ^$^*P* < 0.05, ^$$$^*P* < 0.001 vs. OVX + E2; ^%%^*P* < 0.01, ^%%%^*P* < 0.001 vs. I/R; ^&&^*P* < 0.01, ^&&&^*P* < 0.001 vs. OVX + I/R; ^@@@^*P* < 0.001 vs. OVX + E2 + I/R; ^^^^^*P* < 0.001 vs. Ad-EGFP + I/R). **e** SERCA2a protein expression in vitro (n = 3; ^*^P < 0.05, **P < 0.01, ***P < 0.001 vs. con; ^#^*P* < 0.05, ^##^*P* < 0.01, vs. H/R; ^$^*P* < 0.05, ^$$^*P* < 0.01 vs. E2 + H/R; ^%%^*P* < 0.01 vs. Ad-EGFP + H/R; ^&&^*P* < 0.01, ^&&&^*P* < 0.001 vs. Ad-SERCA2a + H/R; ^@@^*P* < 0.01 vs. si-NC + H/R; ^^^^^*P* < 0.001 vs. si-SERCA2a + H/R; ^aaa^*P* < 0.001 vs. Ad-SERCA2a + E2 + H/R)

First, we confirmed that transfecting Ad-SERCA2a and Ad-EGFP at an MOI of 200 pfu was safe and efficient (Fig. 1a). In vitro, SERCA2a protein levels were significantly increased after incubation with 10 nM E2 compared with the control (con) group (*P* < 0.001) (Fig. 1b). In rats that received intramyocardial injections with Ad-SERCA2a or Ad-EGFP, SERCA2a mRNA and protein expression in cardiomyocytes increased in the Ad-SERCA2a group (*P* < 0.001), but was unchanged in the Ad-EGFP group (Fig. 1c).

In vivo, SERCA2a protein levels were significantly decreased in the OVX and I/R groups compared with the sham group (both *P* < 0.001; Fig. 1d). In contrast, SERCA2a protein levels were increased in the Ad-SERCA2a + I/R group (*P* < 0.05). Compared with the I/R group, SERCA2a expression was lower in the OVX + I/R group (*P* < 0.01). SERCA2a expression in the OVX + E2 + I/R and Ad-EGFP + I/R groups were not statistically distinct from the I/R group.

In vitro, SERCA2a expression was decreased in both the H/R and si-SERCA2a + H/R groups compared with the con group (both *P* < 0.001; Fig. 1e). Nevertheless, the E2 + H/R group had increased SERCA2a expression compared with the H/R group (*P* < 0.05). SERCA2a was also significantly upregulated in the Ad-SERCA2a + E2 + H/R group, while there was no difference in its protein levels between the si-SERCA2a + E2 + H/R group and the si-SERCA2a + H/R group.

### E2 treatment relieved ER stress during MI/R injury by upregulating SERCA2a

**Fig. 2:**
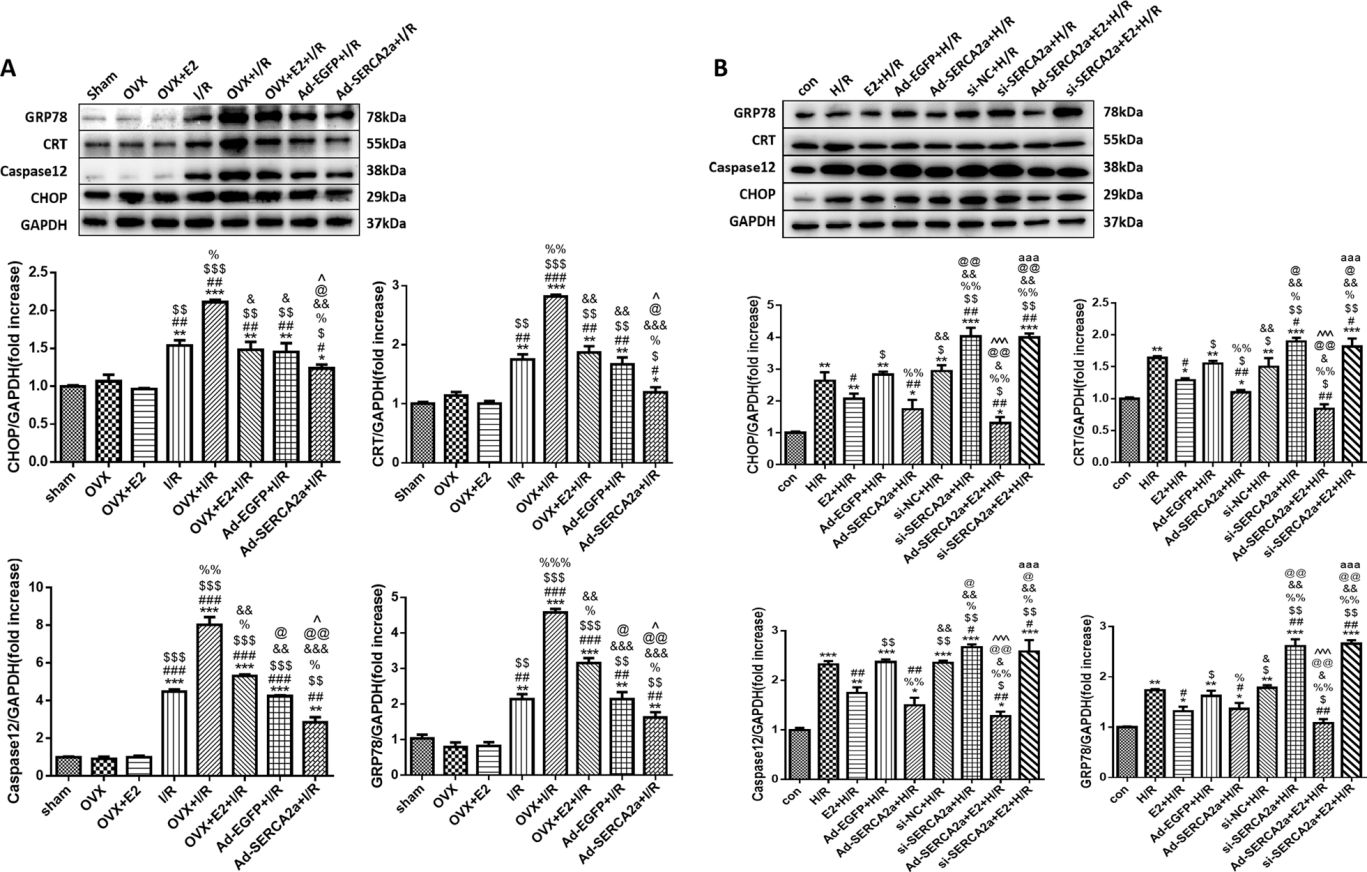
E2 reduced ER stress-related proteins by upregulating SERCA2a. **a** Protein levels of CHOP, CRT, caspase 12, and GRP78 in vivo (n = 3; *P < 0.05, **P < 0.01, ***P < 0.001 vs. sham; ^#^*P* < 0.05, ^##^*P* < 0.01, ^###^*P* < 0.001 vs. OVX; ^$^*P* < 0.05, ^$$^*P* < 0.01, ^$$$^*P* < 0.001 vs. OVX + E2; ^%^*P* < 0.05, ^%%^*P* < 0.01, ^%%%^*P* < 0.001 vs. I/R; ^&^*P* < 0.05, ^&&^*P* < 0.01, ^&&&^*P* < 0.001 vs. OVX + I/R; ^@^*P* < 0.05, ^@@^*P* < 0.01 vs. OVX + E2 + I/R; ^^^*P* < 0.05 vs. Ad-EGFP + I/R). **b** Protein levels of CHOP, CRT, caspase 12, and GRP78 in vitro (n = 3; *P < 0.05, **P < 0.01, ***P < 0.001 vs. con; ^#^*P* < 0.05, ^##^*P* < 0.01 vs. H/R; ^$^*P* < 0.05, ^$$^*P* < 0.01 vs. E2 + H/R; ^%^*P* < 0.05, ^%%^*P* < 0.01 vs. Ad-EGFP + H/R; ^&^*P* < 0.05, ^&&^*P* < 0.01 vs. Ad-SERCA2a + H/R; ^@^*P* < 0.05, ^@@^*P* < 0.01 vs. si-NC + H/R; ^^^^^*P* < 0.001 vs. si-SERCA2a + H/R; ^aaa^*P* < 0.001 vs. Ad-SERCA2a + E2 + H/R)

Increased expression of CHOP, CRT, caspase 12, and GRP78, which are ER stress-related proteins, is commonly used to detect ER stress [37–39]. In vivo, all the groups that underwent MI/R showed increased CHOP, CRT, caspase 12, and GRP78 expression (Fig. 2a). The OVX + I/R group had the most significant increase in the expression of these proteins. Meanwhile, these proteins showed decreased expression in the OVX + E2 + I/R and Ad-SERCA2a groups compared with the OVX + I/R group.

In vitro, H/R caused increased expression of the ER stress-related proteins, especially in the si-SERCA2a + H/R group (Fig. 2b). In the E2 + H/R and Ad-SERCA2a groups, these proteins showed decreased expression compared with the H/R group. However, there were no significant differences between the si-SERCA2a + H/R group and the si-SERCA2a + E2 + H/R group.

### E2 reduced cardiomyocyte apoptosis during MI/R by upregulating SERCA2a

**Fig. 3:**
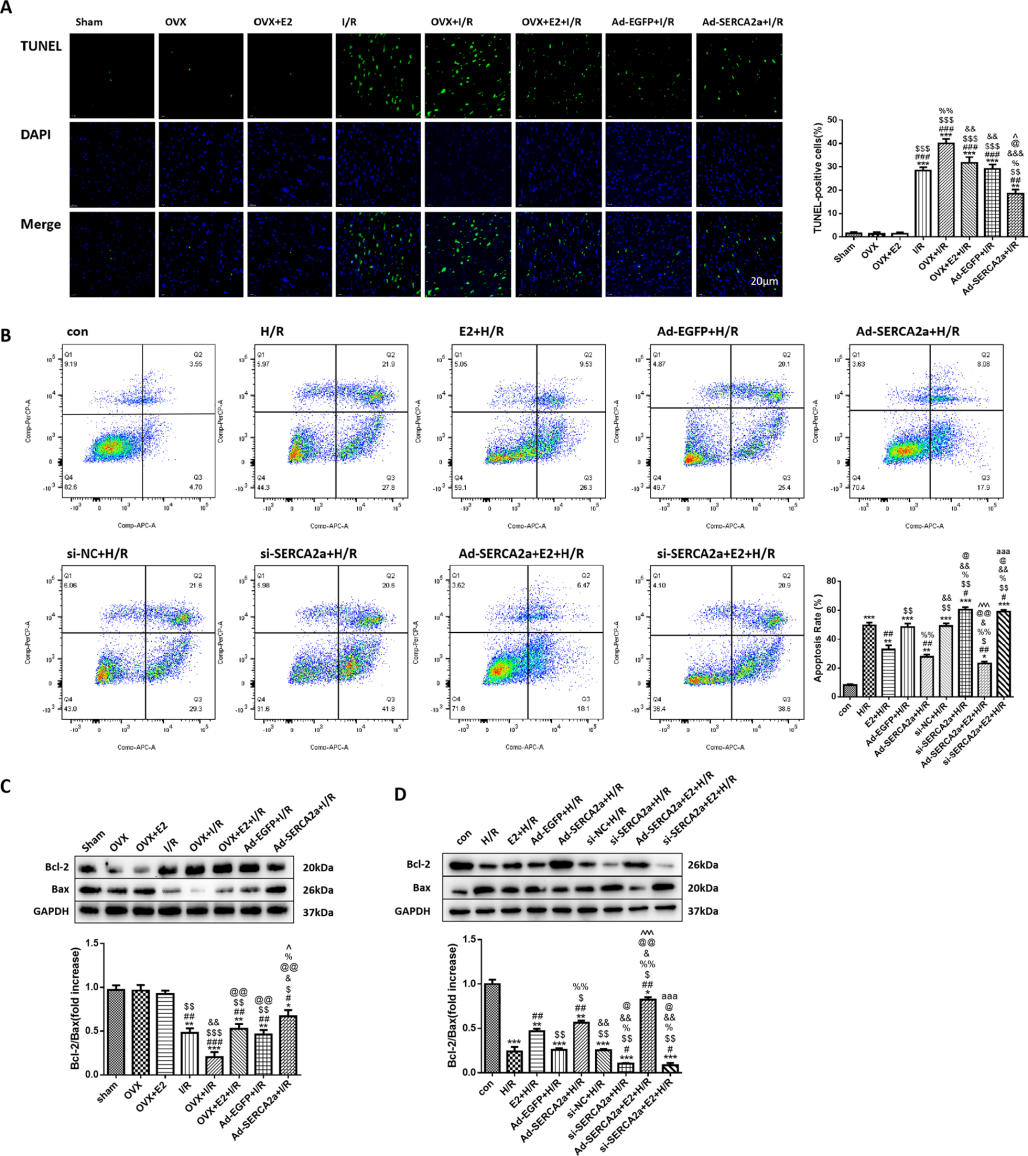
E2 relieved apoptosis by upregulating SERCA2a. **a** Representative images of TUNEL-stained cardiomyocytes of each group in vivo (× 400) and the percentage of TUNEL-positive cells (n = 4). Blue: DAPI-stained nuclei; green: fragmented DNA. **b** Flow cytometry by staining with Annexin V-APC/PI and the apoptosis rate of each group in vitro (n = 4). **c** Bcl-2/Bax ratio in myocardial tissue of rats (n = 3; *P < 0.05, **P < 0.01, ***P < 0.001 vs. sham; ^#^*P* < 0.05, ^##^*P* < 0.01, ^###^*P* < 0.001 vs. OVX; ^$^*P* < 0.05, ^$$^*P* < 0.01, ^$$$^*P* < 0.001 vs. OVX + E2; ^&^*P* < 0.05, ^&&^*P* < 0.01, vs. I/R; ^@@^*P* < 0.01 vs. OVX + I/R; ^%^*P* < 0.05 vs. OVX + E2 + I/R; ^^^*P* < 0.05 vs. Ad-EGFP + I/R). **d** Bcl-2/Bax ratio in NRCMs (n = 3; *P < 0.05, **P < 0.01, ***P < 0.001 vs. con; ^#^*P* < 0.05, ^##^*P* < 0.01 vs. H/R; ^$^*P* < 0.05, ^$$^*P* < 0.01 vs. E2 + H/R; ^%^*P* < 0.05, ^%%^*P* < 0.01 vs. Ad-EGFP + H/R; ^&^*P* < 0.05, ^&&^*P* < 0.01 vs. Ad-SERCA2a + H/R; ^@^*P* < 0.05, ^@@^*P* < 0.01 vs. si-NC + H/R; ^^^^^*P* < 0.001 vs. si-SERCA2a + H/R; ^aaa^*P* < 0.001 vs. Ad-SERCA2a + E2 + H/R)

In vivo, a higher percentage of TUNEL-positive cells means that there was more apoptosis in the tissue (Fig. 3a). Apoptotic cells were apparent in every group that underwent the MI/R protocol. Apoptosis levels were highest in the OVX + I/R group; however, the OVX + E2 + I/R and Ad-SERCA2a + I/R groups had less apoptosis than the OVX + I/R group (*P* < 0.01 and *P* < 0.001, respectively). Meanwhile, each group showed different levels of the apoptosis-related proteins Bcl-2 and Bax (Fig. 3c). The group with the lowest Bcl-2/Bax ratio had the greatest levels of apoptosis. The ratio in every I/R group was lower than that in the sham group, especially for the OVX + I/R group. However, the Bcl-2/Bax ratio was higher in the OVX + E2 + I/R and Ad-SERCA2a + I/R groups than in the OVX + I/R group (both *P* < 0.01).

In vitro, apoptosis rates were determined by apoptosis level of NRCMs (Fig. 3b), and the Bcl-2/Bax ratio (Fig. 3d). H/R increased the apoptosis rate of NRCMs. The si-SERCA2a + H/R group showed more apoptotic cells than the H/R group (*P* < 0.05). Moreover, the apoptosis rate was decreased in the E2 + H/R and Ad-SERCA2a + H/R groups compared with the H/R group (both *P* < 0.01). However, the si-SERCA2a + E2 + H/R group had a similar apoptosis rate to the si-SERCA2a + H/R group. The Bcl-2/Bax ratios of each group showed similar trends as the apoptosis rates.

### E2 alleviated myocardial infarction and cTn I level by upregulating SERCA2a

**Fig. 4:**
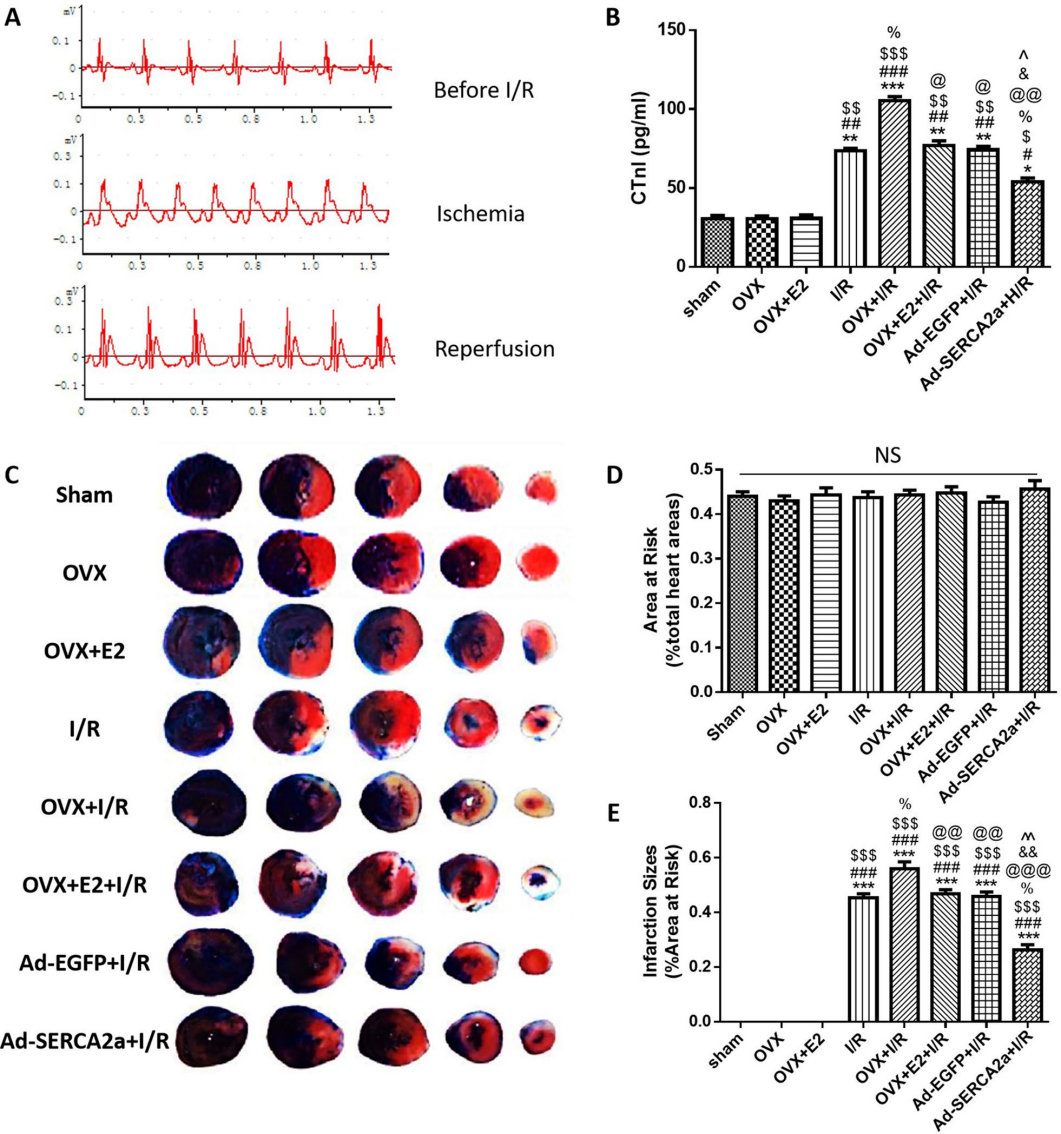
E2 alleviated myocardial infarction and reduced cTn I level by upregulating SERCA2a. **a** The electrocardiography of rats during the establishment of the MI/R model. **b** CTn I levels of each group (n = 3). **c** Representative photographs of Evans blue/TTC-stained heart sections in each group. **d** Area at risk of each group. **e** Percentage of infarction size of each group (n = 3). **d** *P < 0.05, **P < 0.01, ***P < 0.001 vs. sham; ^#^*P* < 0.05, ^##^*P* < 0.01, ^###^*P* < 0.001 vs. OVX; ^$^*P* < 0.05, ^$$^*P* < 0.01, ^$$$^*P* < 0.001 vs. OVX + E2; ^%^*P* < 0.05 vs. I/R; ^@^*P* < 0.05, ^@@^*P* < 0.01, ^@@@^*P* < 0.001 vs. OVX + I/R; ^&^*P* < 0.05, ^&&^*P* < 0.01 vs. OVX + E2 + I/R; ^^^*P* < 0.01, ^^^^*P* < 0.01 vs. Ad-EGFP + I/R

In Fig. 4a, successful ligation of the LAD was confirmed by electrocardiography evidence. The ST elevation following LAD ligation returned during reperfusion. As shown in Fig. 4c–e, the percentages of AAR in each group were similar. I/R resulted in myocardial infarction, and the infarct size was increased in the OVX + I/R group compared with the I/R group (*P* < 0.05). Moreover, the infarct size was decreased in the OVX + E2 + I/R and Ad-SERCA2a + I/R groups compared with the OVX + I/R group (*P* < 0.01, *P* < 0.001, respectively).

Serum cTn I levels in each group were consistent with the infarct size (Fig. 4b).

### E2 increased SERCA2a expression and improved the EF% of the rat heart

**Fig. 5:**
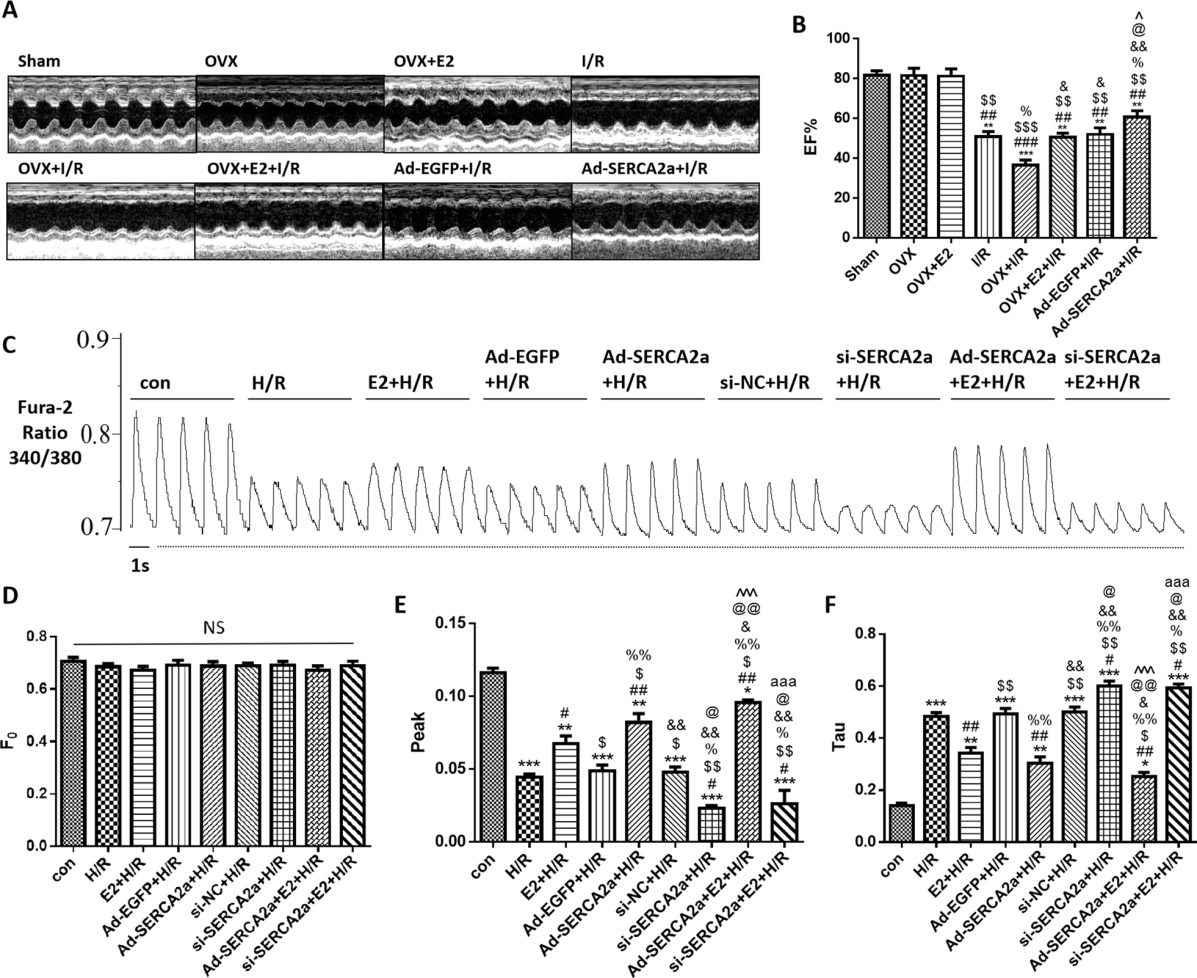
E2 ameliorated EF% and Ca^2+^ transient by increasing SERCA2a expression. **a** M-mode of echocardiography of each group. **b** EF% of the rats in each group (n = 4; **P < 0.01, ***P < 0.001 vs. sham; ^##^*P* < 0.01, ^###^*P* < 0.001 vs. OVX; ^$$^*P* < 0.01, ^$$$^*P* < 0.001 vs. OVX + E2; ^%^*P* < 0.05 vs. I/R; ^&^*P* < 0.05, ^&&^*P* < 0.01 vs. OVX + I/R; ^@^*P* < 0.05 vs. OVX + E2 + I/R; ^^^*P* < 0.01 vs. Ad-EGFP + I/R). **c** Wave of the ratio 340/380 nm in Ca^2+^ transient of each group in vitro. **d**–**f** The trend of the resting intracellular Ca^2+^ levels (F0), maximal value of intracellular Ca^2+^ transient (Peak), and the diastolic intracellular Ca^2+^ transient decay time constant (Tau) (n = 5; *P < 0.05, **P < 0.01, ***P < 0.001 vs. con; ^#^*P* < 0.05, ^##^*P* < 0.01 vs. H/R; ^$^*P* < 0.05, ^$$^*P* < 0.01 vs. E2 + H/R; ^%^*P* < 0.05, ^%%^*P* < 0.01 vs. Ad-EGFP + H/R; ^&^*P* < 0.05, ^&&^*P* < 0.01 vs. Ad-SERCA2a + H/R; ^@^*P* < 0.05, ^@@^*P* < 0.01 vs. si-NC + H/R; ^^^^^*P* < 0.001 vs. si-SERCA2a + H/R; ^aaa^*P* < 0.001 vs. Ad-SERCA2a + E2 + H/R)

I/R injury caused a decrease in the EF% of the heart (Fig. 5a, b). The OVX + I/R group had a lower EF% than the I/R group (*P* < 0.05). However, the OVX + E2 + I/R group showed no difference in EF% from the I/R group. The EF% of the Ad-SERCA2a + I/R group was higher than that of the I/R group (*P* < 0.05).

### E2 ameliorated Ca^2+^ transient by increasing SERCA2a expression

In cardiomyocytes, a transient rise in cytosolic Ca^2+^ concentration initiates contraction [40]. Ca^2+^ transient can reflect the contraction capacity of cardiomyocytes (Fig. 5c). There were no differences in resting intracellular Ca^2+^ levels (F_0_) among all groups (Fig. 5d). H/R caused a decrease in the maximum intracellular Ca^2+^ transient (peak) (Fig. 5e). Compared with the H/R group, the peak value of the si-SERCA2a + H/R group was lower (*P* < 0.05), while the E2 + H/R and Ad-SERCA2a + H/R groups were higher (*P* < 0.05 and *P* < 0.01, respectively). Moreover, the si-SERCA2a + E2 + H/R group was not significantly different from the si-SERCA2a + H/R group. The diastolic intracellular Ca^2+^ transient decay time constant (Tau) was prolonged in the H/R group compared with that in the con group (*P* < 0.001; Fig. 5f). Compared with the H/R group, Tau was increased in the si-SERCA2a + H/R group (*P* < 0.05). Meanwhile, Tau was decreased in the E2 + H/R and Ad-SERCA2a + H/R groups compared with the H/R group (both *P* < 0.01). Nevertheless, the si-SERCA2a + E2 + H/R group was not distinct from the si-SERCA2a + H/R group.

### E2 relieved pathological myocardial damage, fibrosis, and ER swelling via increasing SERCA2a expression

**Fig. 6:**
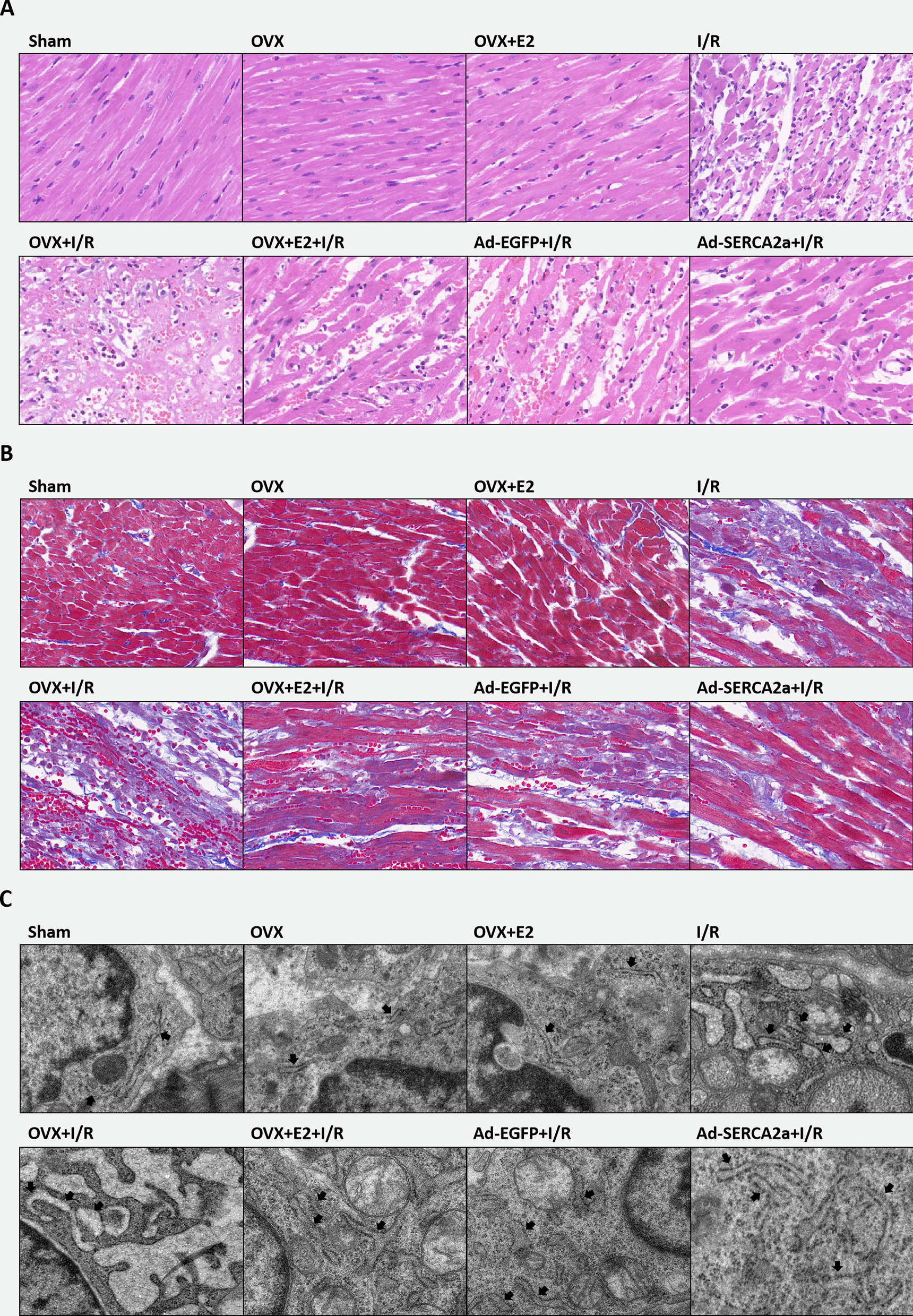
E2 relieved pathological myocardial damage, fibrosis, and ER swelling via increasing SERCA2a expression. **a** Cardiomyocytes HE staining of each group (n = 5). **b** Masson staining of every group (n = 5). Collagenous fibers showed as blue. **c** Representative TEM images of cardiomyocytes (× 11,000; n = 3). Black arrows indicated the ER

As shown in Fig. 6a, cardiomyocytes in the Sham, OVX, and OVX + E2 groups were neatly arranged with normal morphologies and uniform staining. Nuclei were located in the center of myocardial fibers and the nucleolus was apparent. Cardiomyocytes in the I/R group were irregularly and disorderly arranged. Some cases showed edema with fuzzy and pyknotic nuclei. Additionally, myocardial interstitial edema, inflammatory cell infiltration, and local degradation of myocardial tissue was observed. This damage was more severe in the OVX + I/R group than in the I/R group. Some cells were dissolved, necrotic, and fused, with obvious edema, and had reduced nuclei. E2 treatment relieved this damage compared with the OVX + I/R group. Myocardial damage was less severe in the Ad-SERCA2a + I/R group than in the I/R group.

Masson staining was used to observe myocardial fibrosis. Collagenous fibers in myocardial tissue were blue (Fig. 6b). Fibrosis was severe in each I/R group, especially in the OVX + I/R group. The OVX + E2 + I/R and Ad-SERCA2a groups had fewer collagenous fibers than the OVX + I/R group.

As shown by TEM analysis, the ER was extended and swollen in the OVX + I/R group. The OVX + E2 + I/R and Ad-SERCA2a + I/R groups exhibited reduced ER swelling compared with the OVX + I/R group (Fig. 6c).

## Discussion

This study established the MI/R model in vivo and H/R model in vitro to verify that estrogen could increase SERCA2a expression, inhibit ER stress, and alleviate MI/R injury. We confirmed the causal relationship between increased SERCA2a expression and decreased ER stress through adenoviral infection and siRNA transfection of SERCA2a. These results indicated that estrogen has a protective effect on the myocardium by promoting SERCA2a expression.

Our experimental results showed that SERCA2a expression was significantly reduced in the myocardium of rats after bilateral OVX. However, after supplementing E2, SERCA2a protein level almost returned to normal. This result indicates that estrogen has a significant influence on SERCA2a expression in cardiomyocytes. Previous experiments in our laboratory showed that MI/R cause decreased SERCA2a expression in cardiomyocytes [31, 41, 42], which was further confirmed in this study. We also found that SERCA2a expression was more significantly decreased in the OVX + I/R group than in the I/R group. This result may be the superimposition of the effects of OVX and I/R on SERCA2a, but it does not rule out the possibility that after bilateral OVX, the myocardium, which has now lost the protective effects of estrogen, is more sensitive to I/R injury, causing a more severe decrease in SERCA2a expression. After transfection with Ad-SERCA2a, SERCA2a expression was significantly increased, especially at the transcript level. Although I/R caused decreased SERCA2a expression, SERCA2a levels were higher in the Ad-SERCA2a + I/R group than in the sham group. Transfecting si-SERCA2a resulted in a significant decrease of SERCA2a expression in NRCMs. Even with E2 intervention, SERCA2a levels did not increase. This indicated that E2 might affect the expression of SERCA2a at the transcription level.

ER stress is one of the crucial mechanisms of myocardial I/R injury [37–39, 43]. We determined ER stress levels in each experimental group by detecting the levels of ER stress-related proteins, including GRP78, CRT, and CHOP, all of which are increased under ER stress [44]. We found no significant difference in the non-I/R group, indicating OVX does not cause severe ER stress in cardiomyocytes. Under I/R, the ER stress-related proteins increased sensitively and significantly. Among these groups, ER stress in the OVX + I/R group was most severe; however, it was relieved by E2 treatment. This result indicated that E2 could inhibit ER stress during myocardial I/R. The effect of overexpressing SERCA2a on ER stress was consistent with E2 intervention. ER stress was more severe in the si-SERCA2a + H/R group than in the H/R group due to the lack of SERCA2a. However, ER stress was not relieved in the si-SERCA2a + E2 + H/R group. This indicated that the ability of estrogen to relieve ER stress was related to its ability to increase SERCA2a expression.

ER stress can induce apoptosis [21, 45]. Unlike the death receptor signaling pathway or mitochondrial pathway, apoptosis caused by ER stress specifically requires caspase 12 activation [46]. Therefore, we examined the expression of caspase 12 to observe ER stress-related apoptosis. The results showed that caspase 12 expression was consistent with that of ER stress-related proteins. We also demonstrated that the OVX + I/R group in vivo and the si-SERCA2a + H/R group in vitro showed more apoptotic cells along with experiencing severe ER stress. E2 administration and Ad-SERCA2a transfection could reduce apoptosis levels. TUNEL assays and flow cytometric analyses also showed similar results.

The protective effect of estrogen on the myocardium was also reflected in the area of ​​myocardial infarction, serum cTn I levels, and EF%. Meanwhile, conditions that caused more severe ER stress were associated with more pronounced morphological changes to the ER, which showed varying degrees of swelling and expansion.

Currently, there have been few studies focused on the effects of estrogen on SERCA2a expression in cardiomyocytes. However, the results of this study consistently showed that estrogen promoted SERCA2a expression [32, 47–52]. SERCA2a is an essential protein that maintains intracellular Ca^2+^ homeostasis and is a crucial protein for cell excitation–contraction coupling [28]. Decreased SERCA2a expression in cardiomyocytes is one of the leading causes of calcium overload in MI/R injury [53]. Disturbing the Ca^2+^ environment is also crucial for generating ER stress [20]. Therefore, we hypothesized that increasing SERCA2a expression would decrease the ER stress of cardiomyocytes during I/R. This hypothesis was confirmed in this study. Estrogen also affects other calcium handling proteins in cardiomyocytes, such as L-type Ca^2+^ channel, ryanodine receptor, and sodium-calcium exchanger [54]. The roles of estrogen are pervasive, and its protective effects on the cardiovascular system have been previously confirmed [55–57]. The inhibition of ER stress by estrogen has been studied in many diseases related to the nervous system and reproductive system [58, 59], but not in cardiomyocytes. ER stress is an important mechanism of CHD [60]. This study confirmed the inhibitory effect of estrogen on ER stress during MI/R injury. We found that overexpressing SERCA2a can effectively inhibit ER stress. After transfecting si-SERCA2a, even E2 intervention could not relieve the ER stress and cell damage of H/R. Therefore, we believe that part of estrogen's inhibitory effect on ER stress is through increasing SERCA2a expression.

We found that levels of ER stress-related proteins in the OVX + E2 + I/R group were higher than in the I/R group. We suspect that this might because the E2 supplementation did not fully reach the normal levels in rats due to the absorption efficiency of subcutaneous injection. Hormones secreted by the ovaries include estrogen, progesterone, androgens, and other polypeptide hormones and growth factors. Estrogens include estrone, estradiol, and estriol, of which estradiol has the highest activity [61]. We only supplemented with estradiol, but after removing the ovaries, other ovarian hormones were also decreased in the rats. This may be why simply supplementing E2 could not completely restore the MI/R injury of rats to normal.

Previous studies have shown that estrogen can exert myocardial protection through various mechanisms. Estrogen could inhibit nuclear factor-κB (NF-κB), reducing the infarcted area and neutrophil accumulation [62, 63]. It also acts on PI3K/Akt pathway to reduce oxidative stress, ATP depletion, mPTP opening and increase the eNOS activity [64–66]. Additionally, other reports indicated that estrogen protect against I/R injury by activation of the mitochondrial and sarcolemma ATP-sensitive K^+^ channels [13], suppressing Ca^2+^/calmodulin-dependent protein kinase II [54], and modulating manganese superoxide dismutase phosphorylation by mitochondrial p38β at Threonine 79 and Serine 106 [9]. Furthermore, protein kinase C was demonstrated involved in 17β-estradiol-induced protection against MI/R injury and cardiomyocyte apoptosis [11]. Our experimental results further explained the mechanism of myocardial protection of estrogen.

Some downstream pathways of ER stress are also affected by E2. First of all, one of the essential effects of ER stress is to induce specific apoptosis, which has a characteristic increased expression of CHOP and caspase 12. Our experimental results confirmed it. Guo et al. proved that E2 enhances the induction of GRP78 and inhibits the activation of caspase 12 and caspase 3 [24]. It was reported that the anti-apoptotic effect of E2 is dependent on estrogen receptor-mediated upregulation of the expression of anti-apoptotic proteins Bcl-2 and Bcl-xL [67]. Estrogen also rescued pancreatic β-cells from high glucose-induced apoptosis via decreased ER stress followed by downregulation of CHOP [23]. Studies have found that ER stress and inflammatory signaling pathways are coupled through a variety of mechanisms, including NF-κB, activator protein 1 (AP1), reactive oxygen species (ROS), and the release of Ca^2+^ in the ER [68, 69]. Each of the three pathways of UPR can activate NF-κB through different mechanisms [70]. It has been proven that estrogen can inhibit NF-κB [71], such as in I/R injury [72], atherosclerotic tissues [73], lung cancer [74], and other diseases (Additional file 1).

Moreover, autophagy is one of the effective mechanisms proposed for UPR in recent years. Autophagy is the process of cellular self-degradation, which occurs at a low basal level and helps to remove damaged organelles, cytoplasmic proteins, and pathogens. However, various stress conditions, such as hypoxia, may amplify the incidence of autophagy and destroy cell homeostasis, leading to cell death and apoptosis through excessive self-digestion [75]. ER stress signals can lead to an increase in general autophagy [76]. Some studies have shown that estrogen can induce autophagy through specific pathways to protect cells or tissues from damage [77, 78]. However, other studies demonstrated that estrogen could suppress autophagy activation [79–82]. Whether E2 exerts a protective effect through autophagy remains unknown in our experiments. We confirmed that E2 could promote the expression of SERCA2a, which is upstream of ER stress. It is also significant to investigate the effects of E2 on the downstream pathways of ER stress.

There are still many problems that need to be solved. For example, the mechanism through which estrogen acts on SERCA2a is unclear. It is known that estrogen acts on specific receptors through genomic and non-genomic signaling mechanisms. Genomic actions of estrogen are mediated by classic nuclear estrogen receptors alpha and beta (ERα and ERβ). It is now recognized that many of the rapid, non-genomic effects of estrogen are mediated by G-protein coupled estrogen receptors (GPER). In direct genomic regulation, the binding of E2 to the estrogen receptors promotes homo/hetero dimers that translocate to the nucleus to bind directly to gene Estrogen Response Elements, regulating gene transcription [83]. Estrogen receptors at the plasma membrane or in the cytosol can interact with non-genomic targets like kinases and scaffolding molecules modulating multiple signal pathways [65]. All the estrogen receptor subtypes are widely expressed in cardiovascular tissues in various species, ranging from mice to humans [8]. The ways that E2 acts on the expression of SERCA2a are currently unknown. We suspect that both genomic and non-genomic pathways are involved, which requires further research.

In addition, the decline of SERCA2a will cause Ca^2+^ disorders and trigger UPR and ER stress, but how changes in the Ca^2+^ environment trigger UPR is currently unknown.

## Conclusions

Our study demonstrated that estrogen alleviates MI/R injury in rats and inhibits ER stress by upregulating SERCA2a. We also confirmed that inhibiting ER stress is one of the mechanisms through which estrogen protects the cardiovascular system. These results suggest an urgent need to study the downstream targets of SERCA2a. Finally, we provided specific references and the basis for further clinical research on the protective effects of estrogen on cardiovascular disease.

## Data Availability

The datasets used/analyzed to support the conclusion of article are available from the corresponding author upon reasonable request.

## Abbreviations

CHD: Coronary heart disease
MI/R: Myocardial ischemia/reperfusion
E2: 17β-Estradiol
SERCA2a: Sarcoplasmic reticulum Ca^2^^+^ ATPase pump
ER: Endoplasmic reticulum
UPR: Unfolded protein response
IRE1α: Inositol-requiring enzyme 1α
PERK: Protein kinase RNA-like ER kinase
ATF: Activating transcription factor
CHOP: CCAAT/enhancer-binding protein homologous protein
SR: Sarcoplasmic reticulum
OVX: Bilateral ovariectomy
NRCMs: Neonatal rat cardiomyocytes
SD: Sprague–Dawley
H/R: Hypoxia/reoxygenation
LAD: Left anterior descending artery
TTC: 2,3,5-Triphenyltetrazolium chloride
AAR: Area at risk
EF%: Ejection fraction
CRT: Calreticulin
NF-κB: Nuclear factor-κB
AP1: Activator protein 1
ROS: Reactive oxygen species
GPER: G-protein coupled estrogen receptors
